# Arabic-language digital interventions for depression in German routine health care are acceptable, but intervention adoption remains a challenge

**DOI:** 10.1038/s41598-024-62196-8

**Published:** 2024-06-12

**Authors:** Hanna Reich, Ulrich Hegerl, Anja Rosenthal, Caroline Allenhof

**Affiliations:** 1 Department of Psychiatry, Psychosomatic Medicine, and Psychotherapy, Depression Research Centre of the German Depression Foundation, University Hospital, Goethe University, Heinrich-Hoffmann-Str. 10, 60528 Frankfurt am Main, Germany; 2 German Depression Foundation, Leipzig, Germany; 3 Johann Christian Senckenberg Distinguished Professorship, Department for Psychiatry, Psychosomatic Medicine, and Psychotherapy, University Hospital, Goethe University, Frankfurt am Main, Germany; 4 European Alliance Against Depression e.V., Leipzig, Germany

**Keywords:** Depression, Health services

## Abstract

Migrants face many barriers to mental health care, such as different cultural concepts of distress, unfamiliar pathways to care, and language. Digital mental health interventions are effective and scalable in multi-language versions. However, their implementation into routine care is in its infancy. Here, we report on the Arabic- and German-language implementation of two digital interventions in Germany: The iFightDepression^®^ website, providing information about depression to the public, and the iFightDepression^®^ tool, offering guided self-management for depression. Our main goal is to gain empirical knowledge about the success of their implementation and provide evidence-based recommendations for improvement. Data for the current analyses stem from convenience samples, utilizing anonymized user logs of the iFightDepression^®^ website and 15.307 user accounts in the iFightDepression^®^ tool. We found that the acceptability (time on page, usage behavior) of both digital interventions was comparable between the two user groups. The website pervasiveness of the target populations was nine times lower among Arab migrants in Germany than Germans (89 vs. 834 unique page views/ 100,000 inhabitants), but the increase in views was superior and sustained over three years. The adoption of the tool was lower among Arabic than German users (conversion rate from invitation to completed registration: 30.8% vs. 59.0%, *p* < 0.001) and appropriateness was challenged as Arabic users reported higher depression severities upon first registration (*p* = 0.027). Our results show that the uptake of digital interventions for migrants requires facilitation and further tailoring to the needs of the target group.

## Introduction

Treatment gaps for mental disorders are painfully present in today’s health care systems^1^. Undertreatment for instance has been documented for people living with depressive disorders in 21 countries, showing that only 1 in 5 people in high-resource and 1 in 27 in low-resource settings receive minimally adequate treatment^2^. In addition, the long time lag between evidence and implementation of innovations into practice exacerbates gaps in health care services^3^. Global challenges, such as the COVID-19 pandemic or mass migration following armed conflicts, place increasing pressure on societies and health care systems to find timely responses and deliver treatment for mental disorders during crisis and in times of increased need^4^. Internet-delivered treatments for mental disorders have existed for at least 20 years and may serve as a vehicle for innovation^5^. They can improve access to care for many and shall pave the way for more equity in healthcare, ensuring that those who have not been engaged with the health care system will have access to high quality care^6^. In this report, we will showcase the implementation of evidence-informed digital interventions for depression (DID) in Arabic language in Germany as a response to a mental health crisis.

The 2015 humanitarian crisis will serve as an example of a mental health crisis calling for imminent action. Applications for asylum peaked as a result of the war in Syria, with Germany currently hosting around 1.3 million forcedly displaced persons, i.e., the most asylum seekers and refugees (ASR) in the European Union^7^. By 2021, over 6 million refugees from Syria had been registered internationally following the armed conflict^8^. Aside from post-traumatic stress disorders (PTSD), depressive disorders are the most frequent mental health disorders among ASR^9^. A recent meta-analysis reported prevalence rates of 31.5% for both PTSD and depressive disorders in ASR^10^, which is considerably higher than in the German general population^11^. Prevalence rates for depressive disorders varied between subgroups of ASR depending on visa status (asylum seekers: 30.1% vs. refugees: 26.7%), origin (Middle East: 28.6%, Asia: 1.9%, Africa: 23.3%, Europe: 35.8%, mixed: 31.8%), and residence (30.1% community vs. 23.5% refugee/ asylum seeker accommodation)^10^. Syrian ASR are a subgroup within the broader picture of over one million adult migrants from Arab states living in Germany (i.e., also considering first-generation labor migrants, persons joining their family from abroad, foreign students)^12^. While little is known about the mental health of this broader group in Germany, we do know that high-income labor migrants originating from Arab states experience higher rates of depressive symptoms compared to non-migrant Arabs in Qatar^13^. Current literature on the association between depression and migration shows that around one in four migrants globally suffer from depression, which exceeds the prevalence of depression reported by community samples in different nations and demonstrates a need for culturally fitting and targeted responses from migrant host nations and their serving clinicians^14^.

Mental health disorders in ASR in particular, and migrants in general, frequently remain untreated^15^. Potential barriers to health services occur at different levels: the patient, the provider, and at the system level^16^. While language may act as a barrier to psychotherapeutic interventions, it is not per se crucial for the successful delivery of mental health interventions^17^. Nonetheless, limited access to interpreting services has been shown to curtail migrant health care throughout Europe^18,19^. In this case, DID may provide an opportunity for overcoming linguistic barriers to health care provision, as they can be offered and scaled up in different language versions^20^. While the effectiveness of DID is well established^21^ and their cost-effectiveness likely^22,23^, their implementation into routine care is still in its infancy. On the one hand there are successful examples of routine care implementations^24^, on the other hand intervention adoption seems to stay below expectations and usage behavior may vary from that reported in controlled trials^25^. And while the DID for ASR seem to be effective for reducing depression in trial settings^26,27^, challenges such as high dropout rates in ASR have been reported^26^ and their implementation into routine health and social care has not yet been systematically studied.

**Figure 1:**
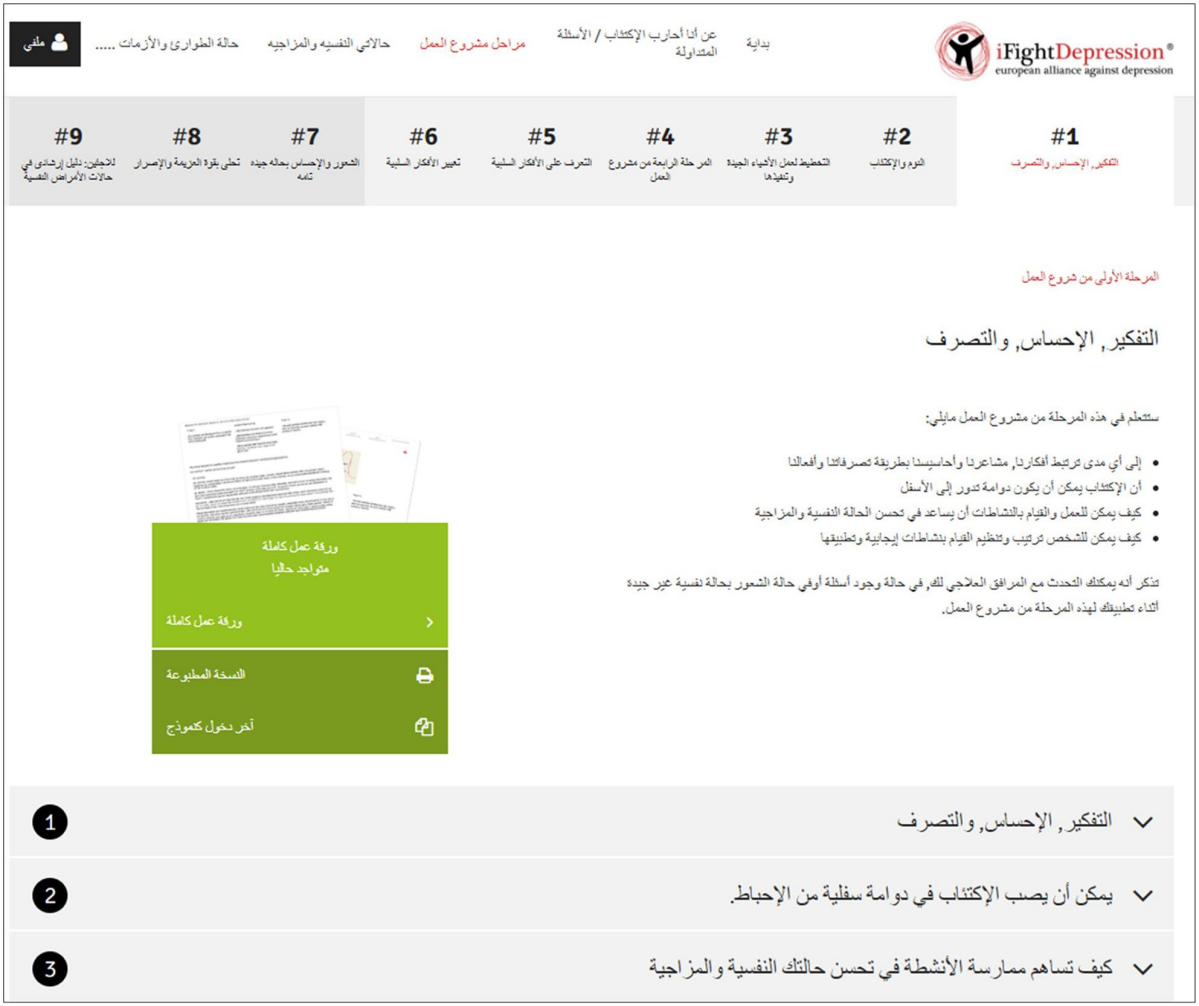
Screenshot of the introduction to the iFightDepression^®^ tool (iFD tool) in Arabic language. The figure gives an impression of the structure of the iFD tool with its six core workshops and the optional workshops (#7—#9). The topics of the workshops are: #1 Thinking, Feeling and Doing, #2 Sleep and Depression, #3 Planning and Doing Enjoyable Things, #4 Getting Things Done, #5 Identifying Negative Thoughts, #6 Changing Negative Thoughts and #7 Feel Better All Round: Healthy Lifestyle. Workshop #8 Finding Inner Strength and #9 Guide to the German Healthcare System, have been developed specifically for ASR. The displayed workshop #1 gives an overview of the content of the tool and an introduction to the activity diary with the options to fill out worksheets online or as a printed version (green field). Below, one can see the option to open informational texts on different subsections of workshop #1 (“1: thinking, feeling and doing”, “2: depression can be a downward spiral”, “3: how activity can make you feel better”). With friendly permission of the European Alliance Against Depression e.V. (EAAD).

In view of the high influx of Arabic-speaking ASR in Germany^28^, the high prevalence of depressive disorders in this group and in migrants in general, and the difficulties to provide imminent and scalable Arabic-language mental health care, Arabic versions of two previously established DID were created: the publicly available iFightDepression^®^ Awareness Website (short: iFD website) and the iFightDepression^®^ tool (short: iFD tool). Both interventions were first developed within an EU-funded project^29^ and have been provided by the European Alliance Against Depression (EAAD) to the public free-of-charge in 19 (iFD website) and 16 (iFD tool) different language versions^30^. The iFD website provides evidence-based information about depression and its treatment for different target groups (e.g., young adults, family and friends, physicians and pharmacists). We consider this to be a crucial step as previous research has shown that the majority of ASR with mental health problems in Europe did not utilize mental health care services^31^. In Syria and neighbouring countries, the explicit labelling of distress as a mental health problem constitutes a source of shame, embarrassment and fear of scandal, with the potential shame extending from patients to their families and affecting the use of mental health services^32^. Providing information about depression to the public may help combat stigma, a main barrier to mental health care. Stigma is negatively associated with help-seeking for mental health related problems; ethnic minorities and males are disproportionally deterred by it^33^. Furthermore, migrants frequently lack knowledge of health services in the host country^34^ and most ASR did not have clear or defined expectations concerning appropriate mental health treatment possibilities^35^. Further, first generation immigrants showed remarkable differences in health care utilization compared to native-born Germans and second generation immigrants: they were less likely to contact medical specialists, but used general practitioners in primary care more frequently^36^. The iFD tool is a guided, web-based intervention for depressive disorders. It provides elements of cognitive behavioural therapy for use in self-management of mild to moderate forms of depression and can be offered in primary care or by mental health care professionals. General practitioners, psychiatrists, or psychotherapists can complete a short CME (Continuing Medical Education) training course to become an iFD tool guide. Once licensed as a guide, they can provide access to the iFD tool for suitable patients. The efficacy of the iFD tool has been established^37^. An expert panel of 12 regional stakeholders and scientists guided the translation into Arabic language and the corresponding cultural adaptation of both interventions. Figure 1 gives an impression of the iFD tool in Arabic language.

This article reports on the implementation of the two Arabic-language DID in public and routine health care in Germany, covering an implementation time span of three years. The main objective is to gain empirical evidence about the success of their implementation in Arabic-speaking compared to German-speaking populations and to provide evidence-based recommendations for improvement. To do so, we seek to answer the following research questions (RQs):

1. How well were the Arabic versions of the iFD website and the iFD tool adopted in the Arabic-speaking population in Germany over time, in relation to the adoption of the German versions?
2. How appropriate was the iFD tool, a DID that was developed for mild to moderate forms of depression, for Arabic-speaking users compared to German-speaking users in Germany?
3. How acceptable was the content of the iFD website and the iFD tool to Arabic-speaking users compared to German-speaking users and compared to health-related content on the internet in general?
4. How much did the iFD website reach the Arabic-speaking population in Germany, in relation to the pervasiveness of the iFD website in the German-speaking population?
5. How sustainable were the implementation activities over time?

Regarding RQ1, we expected that visits to the iFD website would increase in the first two years of implementation, in the case where the iFD website was well adopted by the target group. For the iFD tool, we assessed whether conversion rates from invitation to completed registration differed between Arabic and two different German subgroups of users. In this report, data from these three different iFD tool subsamples were used: Arabic-speaking users (guided use), German-speaking users from routine health care (guided use), and German-speaking users who had registered for the unguided use of the iFD tool during the early days of the COVID-19 pandemic (open use, see below). Regarding RQ2, we assessed whether there were any differences between subgroups of users in depression severity upon first iFD tool registration to determine if the intervention was reaching the intended target population. Regarding RQ3, we assessed whether users spent different amounts of time on pages of the Arabic iFD website compared to the German iFD website and other websites presenting health-related content on the internet. For the iFD tool, we assessed whether there were any differences in usage behaviour within the tool between user subgroups. Regarding RQ4, we assessed whether page views (number of times the webpage was loaded) and unique page views (number of sessions in which this page was viewed at least once) per 100,000 inhabitants in the most current year of implementation differed between the Arabic- and German-speaking populations in Germany. Regarding RQ5 with the aim of gaining an understanding about the sustainability of implementation activities beyond the first two years, we assessed for both language versions whether the page views of the iFD website were still increasing or stable in the third year of implementation compared to the previous years. We regarded the implementation process as sustainable if the activities that have been carried out for the promotion of the digital interventions in the first year after their launch generated longer lasting effects; the continued growth of users or a stable user base in year three of implementation was considered an indicator that the implementation activities have sustained their effect in a digital world of constant change and newness.

## Results

Implementation outcomes and clinical indicators relevant to further implementation will be reported in the following for both DID. For the iFD website, we report on adoption, acceptability, pervasiveness, and sustainability of the intervention. For the iFD tool, we report on adoption, appropriateness, and acceptability. Please refer to Table 1 for implementation indicators for the iFD website and Table 2 for sociodemographic, clinical, and usage behavior characteristics of iFD tool users.

**Table 1:** Implementation indicators for the iFD website by implementation year and language.

		Arabic version		German version	
		Count	% Compared^a^ (%)	Count	% Compared^a^ (%)
Page views (*N*)	1st year	128	–	352,357	–
	2nd year	1092	853	836,196	237
	3rd year	2469	226	855,324	102
Unique page views (*N*)	1st year	97	–	259,825	–
	2nd year	802	827	601,916	232
	3rd year	1708	213	601,887	100
Average time on page (*sec.*)	1st year	118	236	80	160
	2nd year	71	142	77	154
	3rd year	71	142	67	134

The 1st year of implementation started on the 1st of April 2019 for the Arabic version and on the 1st of June 2018 for the German version of the iFD website.

^a^% compared to previous year for page views and unique page views, compared to benchmark of 50 s. for average time on page.

**Table 2:** Sociodemographic, clinical, and usage behavior characteristics for the iFD tool by subsample.

	Subsample			Test statistics	
	Arabic	German (guided)	German (open)	Arabic versus German (guided)	Arabic versus German (open)
Invited users					
Number of active guides	37	919	1 centre^a^		
Number of invited users (n_inv_)	78	7895	13,911		
Invited users per active guide (median)	1	3	–		
Registered users (n_reg_)	24	4661	10,622		
Mean age (s.d., range)	31.0 (9.8, 19–60)	37.3 (13.4, 18–84)	38.0 (12.2, 18–87)	***U* = 41,707, *p* = 0.031, *d* = 0.25 [0.07; 0.42]**	***U* = *172,853*, *p* = .002, *d* = 0.36 [0.14; 0.54]**
n (%) female	5 (20.8%)	2,870 (61.6%)	7,574 (71.3%)	***Chi***^***2***^**(2) = 16.96, *p* < 0.001**	***Chi***^***2***^**(1) = 29.74, *p* < 0.001**
n (%) current psychotherapy	9 (37.5%)	2,522 (54.1%)	3,861 (36.3%)	*Chi*^*2*^(1) = 2.65, *p* = .207	*Chi*^*2*^(1) = 0.01, *p* = .907
n (%) current antidepressant medication	11 (45.8%)	2,245 (48.2%)	3,755 (35.4%)	*Chi*^*2*^(1) = 0.05, *p* = 0.820	*Chi*^*2*^(1) = 1.15, *p* = 0.567
Conversion rate (invitation to registration)	30.8%	59.0%	76.4%	***Chi***^***2***^**(1) = 24.32, *p* < 0.001**	***Chi***^***2***^**(1) = 86.15, *p* < 0.001**
Matching 1: Age and sex					
*n*	24	48	48		
Mean age (s.d., range)	31.0 (9.8, 19–60)	31.0 (9.7, 19–60)	31.0 (9.7, 19–60)		
n (%) female	5 (20.8%)	10 (20.8%)	10 (20.8%)		
Initial PHQ-9 sum score (s.d., range)	16.5 (6.8, 1–27)	12.6 (5.6, 1–23)	12.8 (5.3, 3–23)	***t*(38.99) = 2.41, *p* = 0.027, *g* = 0.64 [0.13; 1.14]**	***t*(37.70) = 2.31, *p* = 0.027, *g* = 0.62 [0.11; 1.12]**
Matching 2: Age, sex, and depression severity					
*n*	24	48	48		
Mean age (s.d., range)	31.0 (9.8, 19–60)	30.6 (10.7, 18–78)	30.7 (9.7, 18–60)		
n (%) female	5 (20.8%)	10 (20.8%)	10 (20.8%)		
Initial PHQ-9 sum score (s.d.)	16.5 (6.8, 1–27)	16.3 (5.8, 3–25)	16.3 (5.4, 7–27)		
Number of completed workshops (s.d.)	0.8 (1.1, 0–4)	1.1 (1.4, 0–6)	0.7 (1.2, 0–6)	*U* = 472, *p* = 0.376, *d* = 0.18 [− 0.08; 0.42]	*U* = 601, *p* = 0.743, *d* = 0.04 [− 0.22; 0.30]

n_inv_ = number of users that have been invited to use the iFD tool or who contacted the German Depression Foundation during the COVID-19 pandemic, n_reg_ = number of users who registered in the iFD tool and completed a baseline assessment. For age, the subsample mean in years is given. PHQ-9 = sum score of Patient Health Questionnaire-9, s.d. = standard deviation. Welch t-tests for unequal variances were applied where necessary, all *p* values were corrected with fdr correction for two tests, as differences concerning one variable were tested in two comparisons each. 95% confidence intervals are provided in [] for effect sizes (d = Cliff’s Delta, g = Hedge’s g). Statistically significant results are printed in bold.

^a^All users who self-registered for the unguided use of the iFD tool during the early days of the COVID-19 pandemic were administered by the coordination centre of the German Depression Foundation in Leipzig, Germany.

### Adoption (iFD website, iFD tool)

Page views and unique page views of the Arabic iFD website increased during the first two years of implementation. The increase from the first to the second year was very steep (853% and 827% of year one’s page views/ unique page views in year two). In the German version, page views and unique page views doubled from year one to year two.

Compared to *N* = 919 registered, active guides using the German version of the iFD tool with their patients in routine health care, only *N* = 37 guides invited users to the Arabic version of the iFD tool. The median number of invited users per guide was one, while guides invited a median of three persons when using the German version. The conversion rate from invitation to completed registration was lower in the Arabic subsample (30.8%) than in both German subsamples (guided: 59.0%, open: 76.4%). The differences were statistically significant (*p* < 0.001).

### Appropriateness (iFD tool)

After matching for age and sex, depression severity upon first registration (PHQ-9) was higher in the Arabic subsample compared to both German groups (*p* = 0.027).

### Acceptability (iFD website, iFD tool)

The mean average time on page was higher for the Arabic version of the iFD website compared to the German version (86.7 s vs. 74.7 s). This difference was not statistically significant (*W* = 5, *p* = 1, Cliff’s delta = 0.11 [− 0.82; 0.88]). The average times on page for both language versions were not statistically different from the benchmark of 50 s. (Arabic version: *V* = 6, *p* = 0.174; German version: *V* = 6, *p* = 0.181).

When matched for age, sex, and initial PHQ-9 sum score, the mean number of completed workshops for the Arabic-speaking subsample was not statistically different from both German-speaking subsamples (*p* ≥ 0.376).

### Pervasiveness (iFD website)

In the most recent year of implementation, there were 945 unique page views from Arabic language users located in Germany, versus 763 unique page views from Arabic language users located outside Germany (not shown in Table 1). That is, 55.3% of Arabic language website users were in Germany. The pervasiveness of the Arabic version of the iFD website in the target population (first generation migrants holding a passport of a country where Arabic is the official language, who were living in Germany at the end of 2021) was 89 unique page views per 100,000 inhabitants.

For the German version of the iFD website, there were 502,863 unique page views from German language users located in Germany, versus 99,024 unique page views from German language users located outside Germany. That is, 83.5% of German language users were in Germany. The pervasiveness of the German iFD website in relation to German passport holders at the end of 2021 in Germany was 834 unique page views per 100,000 inhabitants and therefore, approximately nine times higher than in the Arabic target population.

### Sustainability (iFD website)

However, from year two to year three, page views and unique page views of the Arabic version still more than doubled. Whereas in the German version, page views and unique page views during year three stayed similar to that of year two.

## Discussion

In response to a public health crisis that followed the armed conflict which forced many people to be internationally displaced, two Arabic language DID were translated, culturally adapted, and implemented free-of-charge in Germany. Our report covered a three-year timespan of implementation and compared implementation-related and clinical characteristics of Arabic-speaking users to German-speaking users of both DID.

The publicly available iFD website was well adopted and accepted in Arabic language, with comparable or even exceeding implementation indicators compared to the German language version. However, the pervasiveness within the German-speaking population was around nine times higher than within the Arabic-speaking population in Germany, leaving space for further implementation in this latter population. It is of interest though that the implementation of the Arabic iFD website was even accompanied by an adoption beyond national borders, as 44.7% of unique page views for the Arabic version in the most recent year of implementation originated from outside Germany.

The second DID implemented in Arabic language was the iFD tool, a web-based, guided intervention offering elements of cognitive behavioural therapy as well as information about resilience and the German health care system to individuals with mild to moderate forms of depression. The adoption of the iFD tool stayed thus far below expectations, and conversion rates i.e., the transition from invitation by a guide to user registration and use of the iFD tool, were lower among Arabic-speaking users compared to German-speaking users. Despite the scientific community and public institutions calling, via policy statements and funding opportunities, for low threshold interventions for ASR, only a relatively small number of guides offered the iFD tool to their Arabic-speaking patients. Difficulties in reaching the target populations and managing dropout rates have also been faced by other DID for ASR^38^. The acceptability of the content of the iFD tool was, however, comparable between user groups, even when all subsamples showed a higher depressive symptom burden than the study sample of the efficacy study^37^. Hypothesis about possible barriers to the adoption of this DID among ASR in Germany include, but are not limited to, individual and cultural aspects, as well as aspects concerning the post-migration living situation in Germany, taking into account that the current report investigated the implementation of a DID in regular health and social care (and not a controlled study setting). User behavior in DID may differ greatly when compared between study setting and real-world usage^39^. Apart from stigma associated with mental health problems (see above), a general sense of mistrust as a result of traumatic experiences^40^ might hinder ASR to engage with a guided DID. A lack of technology literacy among ASR has been identified as another challenge associated with using DID^41,42^, but also technological and network issues were causing delay and poor quality of DID^41^ and problems with accessing the internet was identified as a common barrier toward DID in Syrian refugees^42^. These barriers are partially related to housing conditions; most ASR start in transitional housing^43^. In shared accommodation, low privacy whilst using a DID might be a concern as computers for common use are often located in community rooms and bedrooms are frequently shared by multiple persons. As of January 2017, hundreds of thousands of ASR continued to live in temporary shelters after the 2015 influx^44^. A possible solution to this problem might be an app version (instead of the current web-based iFD tool) that could allow content download locally to a mobile phone and less frequent synchronising so that Wi-Fi access and location would be less of a problem when using the iFD tool. Another reason for a low adoption of the iFD tool might be differing help-seeking preferences to what a DID can offer. For instance, a study showed that Arab Australians preferred face-to-face over internet-delivered treatment for psychological distress^45^. Arabic-speaking users of a guided DID in Sweden noted though that a DID in Arabic language was preferrable to face-to-face therapy with an interpreter^46^. Future research needs to assess whether these findings are applicable to migrants from the Arab states living in Germany.

Our straightforward implementation approach of two evidence-informed DID showcased how the research to practice gap can be reduced in the context of a public mental health crisis. Experts guided the translation and cultural adaptation of the previously established DID. The degree of such adaptations proved to be associated with higher efficacy among guided self-help interventions^47^. Some limitations need to be considered though. The DID were evidence-informed, but have not been assessed for efficacy in their Arabic language and culturally adapted versions. There is, however, strong evidence for the efficacy of similar DID in Arabic language^26^ and for the iFD tool itself^37^ including an assessment of possible negative effects^48^. Patients and ASR representatives were not part of the expert group guiding the translation and adaptation process. The parsimonious usage of log-data for the evaluation of the implementation activities did not provide the opportunity to distinguish between residence status of Arabic language users, i.e., we cannot know whether the users of the iFD tool were indeed part of the target group of ASR living in Germany or whether other individuals proficient in the Arabic language were reached. Still, making information about depression available in Arabic language is a first step for supporting ASR in need, as they might be in touch with Arabic speaking migrants in their community who could refer to them the DID by word of mouth if they have used the interventions. However, the significantly lower conversion rates in the Arabic speaking sample point to a specific need to lower the threshold to access to DID in Arabic language. The comparability between the assessed language versions was not fully given as the German iFD website existed in a previous version and therefore, we need to have in mind that it might have started with higher page views and unique page views after its relaunch, whilst the Arabic version was first launched in 2019. The German version was nonetheless chosen as a comparator for the current report for reasons of temporal and spatial proximity to the implementation activities of the Arabic DID in Germany. Other implementation outcomes (e.g., feasibility, fidelity, costs), service outcomes (e.g., effectiveness), and client outcomes (e.g., satisfaction, function) were not assessed. The level of human guidance in digital interventions can induce a significant moderation effect on outcomes^49^, but the fidelity of iFD tool guides regarding their compliance with guidance standards was not assessed and consequently not compared between language versions. We can also not report on the pervasiveness of the interventions within the narrower target group of social and health services for ASR in Germany. Symptom improvement within the iFD tool users has not been assessed due to the small case numbers of Arabic-language users. Finally, the implementation protocol has not been preregistered.

Two DID were implemented in Germany to overcome structural barriers and foster equity in health care access for individuals affected by depression. Whilst there is great general interest in the use of digital health interventions, even countries like Germany, which is relatively far along in the operationalization of necessary frameworks, are struggling with an efficient implementation^50^. Our in-depth analysis of implementation outcomes helped to understand critical points within the implementation process. The analysis of the iFD website’s pervasiveness within the target population showed that there is space for further uptake in the Arabic-speaking community in Germany. Future public relation (PR) activities might want to focus on continuing the promotion of the iFD website in the Arabic-speaking population in Germany (and beyond) to reach similar levels of pervasiveness as with the German-speaking population. An analysis of the findability and an optimization for search machines might be important steps in addition to PR campaigns. Conversion rates in iFD tool users need to be monitored to support guides and invited users in accessing the content of the iFD tool. Next steps may include investigating differences between user groups in accessing the iFD tool and to evaluate the fidelity of different guide groups (medical doctors vs. psychotherapists vs. social workers) with the procedures required by the iFD tool in order to deliver the DID successfully to ASR in routine health and social care. In view of current—including the war against Ukraine—and future societal challenges, the provision of Ukrainian and Russian language versions of the iFightDepression^®^ interventions have been awarded funding by the European Union (EU4Health Programme, Grant Number 101101460) and are already underway. The social return on investment, or, whether the implementation of this publicly available DID was accompanied by increased knowledge about mental disorders, reduced stigma, or even increased help-seeking behaviour among ASR in Germany, remain areas for further research. Modern research designs need to support and drive the successful implementation of innovative, evidence-informed mental health interventions for so-far underserved populations.

## Methods

### Design

This work utilized an effectiveness-implementation type 3 hybrid approach. Hybrid effectiveness-implementation designs were proposed to allow examination of both types of outcomes, i.e., effectiveness and implementation outcomes, within one study instead of using a traditional research pipeline with a sequential approach^51^. The type 3 hybrid focuses primarily on implementation outcomes, while also considering clinical indicators (e.g., symptom severity of users) and usage behaviour within the DID as they relate to the appropriateness and acceptability of the intervention. Following the terminology of implementation outcomes proposed by Proctor and colleagues^52^, we reported on five outcomes (i.e., adoption, appropriateness, acceptability, penetration, and sustainability), each addressed by one RQ (see above). We are using “pervasiveness” throughout the manuscript instead of the term “penetration” that was originally introduced by Proctor and colleagues to avoid any misunderstandings (e.g., due to a confusion with penetration tests as part of the security appraisal for websites). For the operationalization of the main outcomes, please refer to the last paragraph of the introduction section (research questions).

The research was conducted in accordance with the principles of the Declaration of Helsinki. The protocol for the concomitant evaluation of the iFD tool was reviewed and approved by the ethics committee of the Medical Faculty, University of Leipzig on May 27, 2016 (Record Number: 172-16/ek-14032016). Informed consent was obtained from all users of the iFD tool upon registration.

### Study populations

The implementation of the DID aimed to reach adult, native speakers of the Arabic language at two different population levels in Germany: (i) the general public and community, and (ii) patients and individuals with mild to moderate forms of depression. To approximate the target group for Arabic language implementation, the following population estimate was used: at the end of 2021, there were *N* = 1,060,290 adult (≥ 18 years) first generation migrants holding a passport of a country where Arabic is the official language (i.e., Algeria, Bahrain, Chad, Comoros, Djibouti, Egypt, Eritrea, Iraq, Jordan, Kuwait, Lebanon, Libya, Mauretania, Morocco, Oman, Palestine, Qatar, Saudi-Arabia, Somalia, Sudan, Syria, Tunisia, United Arab Emirates, Yemen) and who were living in Germany^12^. As a comparison group, we used the population of *N* = 60,312,535 adults with German nationality that were living in Germany at the same date^53^.

Patients and individuals with mild to moderate forms of depression were identified by primary care and specialist physicians, psychotherapist, or other healthcare professionals and social workers involved in the support of ASR in Germany. These professionals served as guides and invited potential users to the iFD tool. An open, unguided use of the German version of the iFD tool was offered from 18 March to 01 July 2020 only. These users did not receive any face-to-face guidance, but instead received automatic weekly reminder emails during the first six weeks of using the intervention^54^. Users of the iFD tool were included into the analyses when they were at least 18 years old and had given informed consent to participate in the ongoing evaluation of the tool, as required at registration. Registered users of the iFD tool in the Arabic subsample were significantly younger (*p* ≤ 0.05) and more likely to be male (*p* < 0.001) than users in both German subsamples. There were no differences between Arabic users and German guided und open users in the frequency of receiving antidepressant medication or psychotherapy (see Table 2).

### Digital interventions

Two DID were offered for free-of-cost use in Germany. The iFD website is publicly available and offers validated information about depression^30^. The iFD tool is a browser based self-management intervention for patients with mild to moderate depression. It offers psychoeducational content and exercises based on cognitive behavioural therapy to support patients in their everyday life. For the iFD tool, users gained access through active registration following an invitation by a guide (see above). The efficacy of the guided, German language version of the iFD tool has been established by a randomized controlled trial^37^.

#### Translation and cultural adaptations

**Figure 2:**
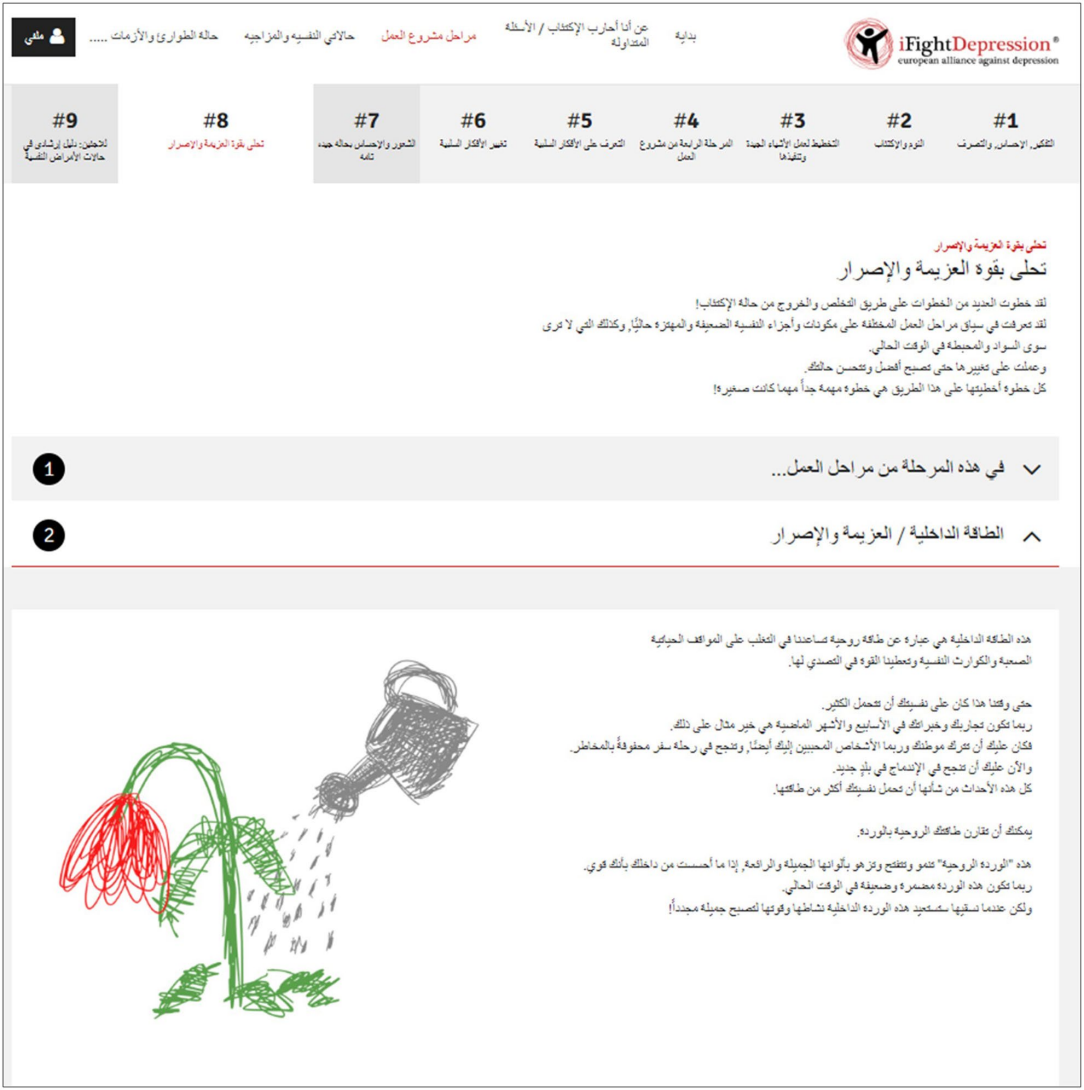
Screenshot of the workshop #8 of the iFightDepression^®^ tool (iFD tool) with content developed for migrants in general and ASR in particular. The figure displays parts of the content of workshop #8 Finding Inner Strength with the subsection. “1: in this workshop” visible and subsection “2: your inner strength” opened up. The following subsections “3: water for my flower” and “4: thoughts for inner strengthening” are not depicted here. This workshop #8 introduces the concept of resilience and inner strength which can be like a flower that sometimes needs watering to get back to full bloom. The workshop is meant to promote a focus on moments where something went well or where difficult situations where successfully mastered and to find the inner strength that was there in these moments to foster resilience and help addressing present challenges. With friendly permission of the European Alliance Against Depression e.V. (EAAD).

Whilst the German DID were developed within an EU-funded project^29^, the Arabic versions were translated and culturally adapted between December 2017 and December 2018. The translation and adaptation process was guided by an expert panel to ensure appropriateness of the DID for ASR. The panel consisted of 12 persons that were either regional stakeholders involved in the social and mental health care for ASR or scientists working in the following fields: affective disorders, migration, treatment of trauma and torture survivors, international or intercultural communications. Based on a literature review, an initial version of the German DID texts was adapted for populations from the Arab states and information regarding health care for ASR in Germany was added. This first version was discussed and improved via face-to-face meetings and group workshops with representatives of the expert panel. The procedure was repeated once more, before a third version was circulated via e-mail to the experts to correct mistakes and create a final version, which was then translated to the Arabic language by a translation agency. Several agencies were considered and two experts selected the agency based on prior experience in medical/psychiatric translations and examples of prior work. The Arabic translations were then entered into the content management system of the DID and piloted with the help of four members of the expert panel to fix bugs and ensure proper functioning. During this process, two additional iFD tool workshops were created enhancing the content of the iFD tool in the Arabic version, one containing exercises on resilience (see Fig. 2) and another one as a guide to the German health care system.

#### Implementation process

The German iFD website was first created on 17 June 2014 and relaunched in its current version on 03 May 2018. The German version of the iFD tool was re-designed after a pilot period and has been available in its current version for routine care use in Germany since 07 October 2016. The Arabic iFD website and iFD tool were launched on 27 March 2019.

Implementation activities were focused on the first year after the launch of the respective DID and language version. The DID were promoted through national and regional press releases, the newsletter of the German Depression Foundation (13,000 subscribers), an information desk at the annual conference of the DGPPN (the largest scientific medical association focusing on mental health in Germany), and social media channels (Facebook, Instagram, LinkedIn, Twitter). For the Arabic DID, bilingual leaflets were created and a large mailing campaign was conducted from May to June 2019, addressing arrival centres, refugee centres, psychosocial centres, and health care centres. Information about the DID was also placed permanently on the websites of the European Alliance Against Depression and the German Depression Foundation.

### Measures

#### iFD website

Indicators for the implementation of the iFD website were inferred from usage data of the website (retrieved via Google Analytics, 28 July 2022). Three 12-month-periods were assessed starting in the month after the creation of the corresponding language version. Indicators for the first year of implementation of the Arabic version were retrieved for 01 April 2019 to 31 March 2020, the second year was from 01 April 2020 to 31 March 2021, and the third year from 01 April 2021 to 31 March 2022. Measures for the German version of the iFD website were created accordingly starting on 01 June 2018 for the first year of implementation and ending on 31 May 2021 for the third year of implementation.

The following indicators were assessed:

- The number of page views and unique page views (i.e., pageviews that were generated by the same user during the same session) per year was used as an indicator for the adoption of the iFD website and to assess the sustainability of implementation activities.
- The average time on page was assessed to understand whether the content of the iFD website was acceptable to users.

#### iFD tool

Indicators for the implementation of the iFD tool were inferred by anonymized log data and self-report data that were routinely collected from invited and registered users (cut-off date: 01 August 2022). Sociodemographic information (age, sex, current treatment for depression; all self-reported) was provided from users during the registration process within the iFD tool. The following implementation indicators were assessed:

- A conversion rate was calculated as the proportion of invited users (n_inv_) in relation to registered users (n_reg_) for each subsample. The conversion rate was used to assess whether the adoption of the iFD tool differed between the Arabic and two German subsamples.
- Depression severity upon first registration was assessed to confirm the appropriateness of the iFD tool for Arabic language users. For this aim, we used the Patient Health Questionnaire-9 (PHQ-9), a short, well-validated, and widely used self-reporting questionnaire assessing symptoms of depression with nine items on a Likert scale ranging from 0 to 3^55,56^. A sum score ranging from 0 to 27 was calculated with higher sum scores indicating more severe depressive symptoms. Completion of the PHQ-9 was mandatory upon registration in the iFD tool.
- The number of completed workshops within the iFD tool served as a proxy for usage behavior and as an indicator for the acceptability of the DID. A workshop in the iFD tool was counted as complete if at least one entry was made in the online worksheet and either all informational texts were opened, or the audio of the informational texts was played (the latter was only available in the German version).

### Statistics

All analyses were conducted with R version 4.2.1^57^. The present analyses had explorative character and as such, shall be understood as a hypothesis-generating contribution rather than confirmatory testing^58^. Each outcome (see Measures section) represented an independent set of hypotheses of possible differences between language versions and subsamples that were using the DID and was tested with alpha = 0.05 (two-tailed). If multiple tests were realized within one outcome, a false discovery rate (fdr) correction was applied.

#### iFD website

Descriptive statistics were reported for page views, unique page views, and average time on page for both language versions and each year of implementation. For each language version, the proportion of page views and unique page views were compared to the previous year of implementation and the differences were reported as percentages (i.e., year two compared to year one; year three compared to year two). The average time on page was not normally distributed (Shapiro–Wilk normality test: *W* = 0.721, *p* = 0.010). For both language versions, the mean average time on page was compared against a benchmark (expected mean) of 50 s using a Wilcoxon Signed Rank Test with continuity correction. This benchmark was the average time on page that was recorded in the year 2020 across multiple countries in the category “health and beauty”^59^. A Mann–Whitney U-test was used to compare the average time on page of the Arabic and German version in the three years of implementation against each other; Cliff’s Delta with its 95% confidence interval is given. Unique page views that were generated by users located in Germany in the most current year of implementation were estimated per 100,000 inhabitants as an indicator of the pervasiveness of the iFD website in the Arabic- and German-speaking populations in Germany. We worked with population estimates as reference data for the usage of the iFD website within the target groups, since it is a publicly available DID and as such, does not record or distinguish between residential status of users.

#### iFD tool

The number of guides who invited users for the Arabic and German versions of the iFD tool and the median number of invited users per guide were assessed. Descriptive statistics for sociodemographic, clinical, and usage behavior characteristics were reported for registered users of the iFD tool. To assess if the data followed a normal distribution, Q–Q plots and histograms were generated for visual inspection and a Shapiro–Wilk test was performed. Differences between subsamples were assessed in all registered users of the Arabic versus both German subsamples using chi-squared tests (categorical data) or two-sample t-tests (continuous data) or Mann–Whitney U-test (non-normal continuous or ordinal data). Cliff’s Delta for Mann–Whitney-U test and Hedges’ g for two sample t-test are given with their 95% confidence intervals.

To cope with the highly varying subsample sizes within the iFD tool, a matching strategy was applied to compare the baseline depression severity between subsamples. To prepare the data, all cases with missing values for age, sex, or the PHQ-9 scores were excluded. From the pool of German-language guided and unguided iFD tool users, samples matched for age and sex to the Arabic-speaking users were generated using propensity scores with nearest neighbor matching procedures from the R-package “MatchIt” and a 1:2 ratio. In a second matching procedure, the same method was applied matching for age, sex, and initial PHQ-9 scores to allow comparison of usage behavior (number of completed workshops).

## Data Availability

The datasets used and analyzed during the current study are available from the corresponding author on reasonable request.
